# Rational Design and Characterization of D-Phe-Pro-D-Arg-Derived Direct Thrombin Inhibitors

**DOI:** 10.1371/journal.pone.0034354

**Published:** 2012-03-23

**Authors:** Ana C. Figueiredo, Cristina C. Clement, Sheuli Zakia, Julian Gingold, Manfred Philipp, Pedro J. B. Pereira

**Affiliations:** 1 IBMC - Instituto de Biologia Molecular e Celular, Universidade do Porto, Porto, Portugal; 2 Department of Chemistry, Lehman College & Biochemistry Program, CUNY Graduate School, New York, New York, United States of America; 3 MD Program at Mount Sinai School of Medicine, New York, New York, United States of America; University of Graz, Austria

## Abstract

The tremendous social and economic impact of thrombotic disorders, together with the considerable risks associated to the currently available therapies, prompt for the development of more efficient and safer anticoagulants. Novel peptide-based thrombin inhibitors were identified using *in silico* structure-based design and further validated *in vitro*. The best candidate compounds contained both l- and d-amino acids, with the general sequence d-Phe(P3)-Pro(P2)-d-Arg(P1)-P1′-CONH_2_. The P1′ position was scanned with l- and d-isomers of natural or unnatural amino acids, covering the major chemical classes. The most potent non-covalent and proteolysis-resistant inhibitors contain small hydrophobic or polar amino acids (Gly, Ala, Ser, Cys, Thr) at the P1′ position. The lead tetrapeptide, d-Phe-Pro-d-Arg-d-Thr-CONH_2_, competitively inhibits α-thrombin's cleavage of the S2238 chromogenic substrate with a K_i_ of 0.92 µM. In order to understand the molecular details of their inhibitory action, the three-dimensional structure of three peptides (with P1′ l-isoleucine (fPrI), l-cysteine (fPrC) or d-threonine (fPrt)) in complex with human α-thrombin were determined by X-ray crystallography. All the inhibitors bind in a substrate-like orientation to the active site of the enzyme. The contacts established between the d-Arg residue in position P1 and thrombin are similar to those observed for the l-isomer in other substrates and inhibitors. However, fPrC and fPrt disrupt the active site His57-Ser195 hydrogen bond, while the combination of a P1 d-Arg and a bulkier P1′ residue in fPrI induce an unfavorable geometry for the nucleophilic attack of the scissile bond by the catalytic serine. The experimental models explain the observed relative potency of the inhibitors, as well as their stability to proteolysis. Moreover, the newly identified direct thrombin inhibitors provide a novel pharmacophore platform for developing antithrombotic agents by exploring the conformational constrains imposed by the d-stereochemistry of the residues at positions P1 and P1′.

## Introduction

Thromboembolic diseases are highly prevalent in industrialized countries and the development of new therapeutic approaches is essential to improve both life quality and expectancy of the patients. Prevention of pathological clot formation can be achieved by specifically inhibiting the serine proteinase α-thrombin, an enzyme that occupies a central role in the blood coagulation cascade, where it plays both pro and anticoagulant roles. The discovery of safe, selective, and orally available anticoagulants has proved to be a challenging endeavor, primarily due to the serious side effects (e.g. bleeding and liver toxicity) of the compounds considered so far, limiting their therapeutic application [1]. Therefore, the development of new synthetic direct thrombin inhibitors (DTI) has been the focus of intense research [2], [3]. DTI inhibit both soluble and fibrin-bound thrombin, have predictable pharmacokinetics and are classified as univalent or bivalent depending on whether they bind exclusively to the active center or simultaneously to the active center and the exosite I of thrombin. Synthetic thrombin inhibitors can also be subdivided into irreversible, reversible covalent or reversible non-covalent [4].

Irreversible thrombin inhibitors include PPACK (d-Phe-Pro-Arg-chloromethylketone) [5] and other halomethylketones [6] that form a covalent tetrahedral acyl intermediate upon binding to thrombin by reaction with the active site residues. Given the low specificity of irreversible inhibitors for thrombin, the search for new anticoagulant therapies has been focused on reversible DTI. Reversible bivalent inhibitors as lepirudin (a variant of the naturally occurring leech inhibitor hirudin) and bivalirudin (or hirulog-1, a synthetic hirudin derivative) [7], [8], as well as the low molecular-weight active-site inhibitor argatroban [9], [10] are among the parentally administered DTI used in clinical settings, namely for the treatment of patients with heparin-induced thrombocytopenia [11], [12]. More recently, the univalent inhibitor dabigatran etexilate, an oral anticoagulant that is rapidly absorbed and converted to its active form dabigatran [13], revealed being a promising alternative to the traditional use of warfarin in stroke prevention in atrial fibrillation [14]. This drug was designed by a structure-driven approach based on a peptidic DTI structure in complex with bovine thrombin [15].

The interest on the development of small peptidomimetic inhibitors (<500 Da), dating back to 1982 with the dipeptide-derived d-Phe-Pro-Agamantin [16], was mostly driven by their potential to traverse the gastrointestinal epithelium and enter the blood circulation. More recent approaches include the study of several synthetic analogs of the angiotensin converting enzyme breakdown product of bradykinin (RPPGF) [17], [18], [19], [20] as well as 1,3,5-trisubstituted benzenes [21] as potent thrombin inhibitors.

Virtual screening by ligand docking is a common approach in modern drug design [22]. In the case of anticoagulant compounds, this is particularly appealing given the abundant experimental structural information on thrombin-inhibitor complexes currently available. This information, together with modern structure-based drug design (SBDD) methods, can be used to shorten the discovery and design phases of new drugs [23].

In this work a SBDD approach was used for designing novel peptidic DTI derived from the d-Phe-Pro-d-Arg-P1′-CONH_2_ (fPr-P1′) tetrapeptide scaffold. As peptides containing d amino acids are less susceptible to proteolytic cleavage [24], the replacement of l-Arg by d-Arg at position P1 was therefore hypothesized to confer resistance to proteolysis, allowing these sequences to function as thrombin inhibitors. The crystallographic structures of the α-thrombin complexes of three lead compounds having l-isoleucine (fPrI), l-cysteine (fPrC) and d-threonine (fPrt) at the P1′ position are reported. The experimental models revealed substrate-like binding of the inhibitors to the active site of the enzyme and provided a structural explanation for their resistance to proteolysis. Furthermore, the extent of the observed interactions established by the residue at position P1′ in the three-dimensional structures of the complexes, correlated well with the experimental binding affinities for all three lead peptidic DTI. Thus, the newly identified sequence d-Phe(P3)-Pro(P2)-d-Arg(P1)-P1′-CONH_2_ emerges as a potential antithrombotic scaffold which can be further optimized at the P3, P2 and P1′ positions to improve its potency, selectivity, bioavailability, and anticoagulant properties.

## Materials and Methods

### Molecular docking and *in silico* structure-based design of peptide libraries

New peptidic non-covalent DTIs were designed by generating peptide lead compounds derived from the substrate sequence Phe(P3)-Pro(P2)-Arg(P1). The free energy of interaction between each ligand and thrombin was calculated with the built-in molecular mechanics force field (MMFF) provided by the docking software *SCULPT* (Accelrys).

During the original screening, the hexapeptides [d-Phe(P3)-Pro(P2)-d-Arg(P1)-P1′-P2′-P3′-CONH_2_] and pentapeptides [d-Phe(P3)-Pro(P2)-d-Arg(P1)-P1′-P2′-CONH_2_] were used as scaffolds for developing the optimized final tetrapeptide lead sequence, d-Phe(P3)-Pro(P2)-d-Arg(P1)-P1′-CONH_2_. Once the lead tetrapeptide scaffold was found to have higher affinity for thrombin than the hexa and pentapeptides, based on structure-activity relationship (SAR) studies on thrombin inhibition conducted *in vitro*, new peptide candidate inhibitors were further designed as derivatives of the tetrapeptide motif d-Phe(P3)-Pro(P2)-d-Arg(P1)-P1′-CONH_2_. New peptide sequences were developed by varying the P1′ positions both with l and d natural or non-natural amino acids, covering a wide range of chemical structures. The protein template used in all molecular docking experiments was the structure of human α-thrombin in complex with the covalent inhibitor PPACK (PDB entry 1ABJ [25]). The new peptide structures were drawn with ISIS Draw 2.2.1 (Accelrys), imported as 3D-structures in *SCULPT*, and manually docked into the active site of thrombin by alignment with the PPACK inhibitor. The resulting thrombin∶peptide complex was minimized using the *SCULPT* built-in molecular mechanics force field (MMFF94). After each round of minimization, the free energy of interaction (scoring function) was assessed using both Van der Waals and electrostatic force fields.

### Peptide synthesis and purification

Peptides were synthesized using standard solid-phase fluorenylmethyloxycarbonyl (Fmoc) chemistry on a 432A Synergy Personal Peptide synthesizer (ABI) as previously described [19]. Amide Rink resin (Novabiochem) was used to produce all peptides as C-terminal amides. A 20% solution of piperidine in N,N′-dimethyl formamide (DMF) was used to remove the Fmoc protecting group from the amide Rink resin linker, and again to remove the Fmoc-protecting group after each coupling cycle. Coupling was performed using a fourfold excess of amino acid and a solution of 0.4 M hydroxybenzotriazole (Advanced Chem Tech) and O-benzotriazole-N,N,N′,N′-tetramethyl-uroniumhexafluoro-phosphate (Advanced Chem Tech) in DMF, in the presence of diisopropylethylamine. Upon synthesis completion, the resin was washed with DMF, dichloromethane, and dried. The peptides were cleaved from the resin and side-chain-protecting groups removed after treatment for 3–4 h with a cleavage cocktail consisting of 50 µL of ethanedithiol, 50 µL of thioanisole and 900 µL trifluoroacetic acid (TFA) and precipitated with cold methyl *tert*-butyl ether. Peptides were solubilized in 50% (v/v) acetonitrile. The filtered crude material was then purified on a C18 reversed-phase column (Phenomenex) using a linear gradient of 0–75% acetonitrile in 0.1% TFA, at a rate of 2% per minute. The identity of each peptide was determined by electrospray ionization (ESI) mass spectrometry run in the positive mode.

### Inhibition of thrombin's hydrolytic activity towards a chromogenic substrate

In a first instance, the inhibitory constants (K_i_) for all peptides were determined using pseudo-first order kinetics. Constant concentrations of bovine α-thrombin (5 nM; Sigma) and of the chromogenic substrate S2238 (H-d-Phenylalanyl-l-pipecolyl-l-arginine 4-nitroanilidedihydrochloride; 2.8 µM, corresponding to 0.7× its K_M_ for thrombin; Chromogenix) were used, with varying concentrations (0–100 µM) of each inhibitor. Substrate hydrolysis at 25°C, in sodium phosphate buffer pH 7.46, with 0.2 M NaCl and 50 µg/ml bovine serum albumin was monitored in real-time at 405 nm using a thermostated spectrophotometer (Cary), assuring that the hydrolysis of the chromogenic substrate was complete. Each kinetic trace was fitted to a first-order reaction mathematical model (At = A_0_*e**^−kt^** or A_t_ = A_0_+a*(1−e**^−kt^**)). The K_i_ was determined from the equation K_i_ = [I]/(k_obs_ uninhibited/k_obs_ inhibited−1), where k_obs_ is the pseudo-first order rate constant and [I] is the molar inhibitor concentration. For the inhibitors that were structurally characterized, the kinetic parameters of S2238 hydrolysis and the type of inhibition were determined using 5 nM bovine α-thrombin (Sigma) and varying concentrations of both substrate (0–200 µM) and peptide inhibitors (0–50 µM). The data was fitted to a competitive enzyme inhibition model using Prism5 (GraphPad Software) and the built-in nonlinear regression global analysis package to determine the values for K_i_, K_M_ and V_max_. The goodness of fit was assessed using the built-in statistical analysis package, which enabled the calculation of R^2^ values.

### Inhibitory activity towards factor Xa and trypsin

Factor Xa (15 nM; American Diagnostica) or trypsin (25 nM; Sigma) was incubated for 15 min at room temperature with 20–1600 µM of fPrI, fPrC or fPrt in 50 mM Tris-HCl pH 8.0 and 150 mM NaCl (factor Xa) or 50 mM Tris-HCl pH 8.0, 150 mM NaCl and 160 mM CaCl_2_ prior to substrate addition. The chromogenic substrates were S2222 (nα-Benzoyl-l-isoleucyl-l-glutamyl-glycyl-l-arginine-4-nitroanilide hydrochloride; American Diagnostica) for factor Xa or BAPNA (nα-Benzoyl-l-arginine-4-nitroanilide hydrochloride; Sigma) for trypsin. Substrate hydrolysis was monitored at 405 (S2222) or 410 nm (BAPNA) using a thermostated spectrophotometer (Cary). IC_50_ values were determined using the built in equation provided by Prism5 (GraphPad Software). Values for the inhibition constant (K_i_) were calculated assuming a competitive mechanism of inhibition according to the equation K_i_ = IC_50_/(1+S/K_M_), where S is the substrate concentration (0.18 mM for S2222 and 1.84 mM for BAPNA) and K_M_ is the Michaelis constant of the substrate (0.3 mM for S2222 and 0.95 mM for BAPNA).

### Thrombin time (TT) assays

Human plasma (800 µl) was mixed with 200 µl of 0–1 mM (final concentration) peptide solutions in 20 mM Tris pH 8.0, 100 mM NaCl. The thrombin time was measured at BM Análises Clínicas following standard protocols.

### Inhibitor resistance to proteolysis

Each peptide inhibitor (20 µM) was incubated with α-thrombin (20 µM) at room temperature in phosphate buffer pH 7.46 with 0.2 M NaCl. After 24 h incubation, aliquots were removed and the reaction was quenched with 0.1% TFA. The samples were analyzed on an ESI spectrometer run in the positive mode. As the expected molecular mass of the tripeptide resulting from the hydrolysis of the D-Arg-P1′ peptide bond is 418.5 Da, spectra were acquired in the 400–1000 Da window.

### Crystallization and data collection

Sample preparation, crystallization, and X-ray diffraction data collection were performed as previously described [26].

### Structure Determination and Refinement

The structure of unliganded human α-thrombin was solved by molecular replacement with Phaser [27] using the coordinates from PDB entry 1VZQ [28] as search model. The refined model of unliganded human α-thrombin was subsequently used as search model in the structural determination of the fPrI, fPrC and fPrt thrombin complexes. The initial electron density difference maps showed interpretable density for all inhibitors.

Cycles of manual model building with Coot [29], alternating with cycles of crystallographic refinement with PHENIX [30], were performed until completion of the models. The models were initially subjected to positional simulated annealing, followed by refinement of TLS parameters (determined using the TLS Motion Determination software [31], as implemented in the TLSMD server [32]) and individual atomic displacement parameters. When most of the solvent structure was built, the inhibitor was fitted to the electron density maps. A sodium cation (in the sodium binding loop), and a chloride and an iodide anion, as well as 2-methyl-2,4-pentanediol (MPD) molecules from the crystallization buffer were also located. An ordered N-acetyl-glucosamine moiety was also modeled, bound to Asn60G. In the final cycles, occupancy of the ions and sugar groups was refined. Individual anisotropic ADP refinement was carried out for the light and heavy chains of thrombin for the higher resolution models (unliganded thrombin, fPrI and fPrt complexes).

To correctly assign the bound halide anions, from the iodide, chloride and bromide present in the crystallization buffer, two datasets for the same crystal were collected at different energies (12 keV and 14 keV). Inspection of the peaks at the anion positions in the anomalous difference maps allowed the unambiguous identification of the bound ions as chloride and iodide.

The final models of unliganded thrombin and of the thrombin∶fPrt complex comprise residues Ile16 to Glu247 and Ala1B to Arg15 of one thrombin molecule (chains H and L, respectively). The model for the thrombin∶fPrI complex comprises residues Ile16 to Glu247 and Ala1B to Ile14K, and that for the thrombin∶fPrC complex comprises residues Ile16 to Phe245 and Ala1B to Ile14K. The models contain one each of sodium, iodide and chloride ions. An N-acetyl-glucosamine sugar moiety is attached to Asn60G in chain H. Three, two or one molecule of MPD from the crystallization buffer were observed in the models of unliganded thrombin, fPrI/fPrt and fPrC, respectively. Residues of loop 148 (Thr147 to Lys149E) were not well defined in the electron density maps and were not included on the final models. Refinement statistics are summarized in Table 1. The refined coordinates and structure factors were deposited at the PDB with accession numbers 3U69, 3U8R, 3U8T and 3U8O.

**Table 1:** Data collection and refinement statistics.

Thrombin structure	Unliganded(a)	fPrI complex(a)	fPrC complex(a)	fPrt complex(a)
**Data collection and processing**				
Space group	P2_1_2_1_2_1_	P2_1_2_1_2_1_	P2_1_2_1_2_1_	P2_1_2_1_2_1_
Unit cell dimensions (Å)	a = 57.5; b = 73.0; c = 83.0	a = 51.6; b = 76.5; c = 83.3	a = 51.8; b = 77.1; c = 83.4	a = 57.5; b = 72.7; c = 83.1
Resolution range (Å)	72.9-1.55 (1.63-1.55)	51.6-1.47 (1.55-1.47)	35.0-1.86 (1.96-1.86)	47.3-1.28(1.35-1.28)
**Refinement**				
Resolution range (Å)	47.3-1.55	43.8-1.47	35.0-1.86	36.3-1.28
Rfactor^b^/Free Rfactor^c^ (%)	13.3/16.7	12.7/15.8	15.4/19.6	12.8/14.8
N° of unique reflections (working/test set)	49654/2508	56347/2860	28541/1414	86922/4376
Water molecules	364	358	265	444
Ions (Na^+^/I^−^/Cl^−^)	1/1/1	1/1/1	1/1/1	1/1/1
Total number of atoms	2795	2706	2608	2938
Average overall B-factor (Å^2^)	21.4	17.4	20.8	17.8
Average protein B-factor (Å^2^)	19.0	15.1	19.3	14.9
Average main chain B-factor (Å^2^)	16.3	12.0	16.1	12.4
Average side chain B-factor (Å^2^)	21.5	18.0	22.4	17.2
Average water B-factor (Å^2^)	34.0	30.4	31.3	31.7
r.m.s.d. bonded Bs (Å^2^)	2.59	3.29	3.97	2.18
r.m.s.d. bond lengths (Å)	0.010	0.010	0.019	0.013
r.m.s.d. bond angles (^°^)	1.29	1.29	1.85	1.47
**Ramachandran plot statistics**				
Residues in allowed regions (%)	100	100	100	100
Residues in favoured regions (%)	96.9	96.9	96.1	97.7
Residues in disallowed regions (%)	0	0	0	0
**Estimated coordinate error**				
E.s.d from Luzzati plot (Å)	0.156	0.145	0.171	0.122
DPI^d^ (Å)	0.086	0.070	0.108	0.048

(a) Values in parenthesis correspond to the outermost resolution shell.

(b) R_factor_ = Σ||F_o_|−|F_c_||/Σ|F_o_| where |F_o_| and |F_c_| are observed and calculated structure factor amplitudes, respectively.

(c) Free R_factor_ is the cross-validation R-factor computed for a randomly chosen subset of 5% of the total number of reflections, which were not used during refinement.

(d) Diffraction-data precision indicator.

## Results and Discussion

### Structure-based design of peptide libraries as potential direct thrombin inhibitors

**Figure 1:**
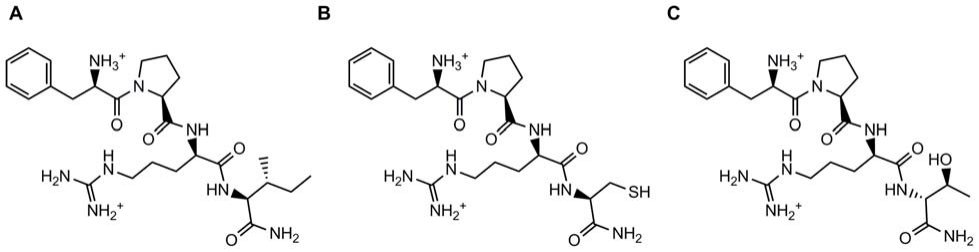
Structure of the peptide inhibitors characterized in complex with human α-thrombin. fPrI (d-Phe-Pro-d-Arg-Ile-CONH_2_), fPrC (d-Phe-Pro-d-Arg-Cys-CONH_2_), fPrt (d-Phe-Pro-d-Arg-d-Thr-CONH_2_).

In an attempt to discover new anticoagulants with lower risk of bleeding, a new generation of peptidic DTI derived from the d-Phe-Pro-d-Arg tripeptide scaffold was developed (Figure 1). Besides the optimal d-Phe-Pro dipeptide at positions P3-P2 [5], the d-isomer of arginine was selected for position P1 in order to improve resistance to proteolytic degradation by thrombin. Given the peptidic nature of the compounds, they would be best suited for intravenous delivery (e.g. in the treatment of acute thrombotic events and in surgical settings) therefore minimizing the possible impact in bioavailability of a basic P1 moiety, as in other similar cases [33]. The peptide libraries generated contained different l- and d-isomers of natural (and some non-natural) amino acids at positions P1′, P1′ and P2′ or P1′, P2′ and P3′, thus generating peptide inhibitors with formulae ranging from d-Phe-Pro-d-Arg-P1′-CONH_2_ to d-Phe-Pro-d-Arg-P1′-P2′-P3′-CONH_2_.

Initial docking experiments allowed a fast screening of the structural fitness between thrombin and the peptide ligands, based on the Van der Waals force field included in the MMFF94 package. The hits were ranked after improvement of the initial predicted relative free energy of interaction-based main scoring function by inclusion of the electrostatic force field. From the very beginning it was clear that the peptides with P2′ or P2′ and P3′ occupancy scored much lower than the tetrapeptidic compounds, and their screening was truncated. One hundred and twenty virtual lead compounds with a predicted protein-peptide ligand binding energy of less than −30.0 kcal/mol (corresponding to low µM to low nM K_i_ values) and reasonable fit to thrombin's active site (as judged by visual inspection) were selected from the more than 1000 virtually screened peptide sequences. The top-ranking 28 compounds (Table 2), which were synthesized for further experimental validation (see below), all contained the d-Phe-Pro-d-Arg-P1′-CONH_2_ sequence, differing only in the chemical nature of the residue at position P1′.

**Table 2:** Inhibition of bovine thrombin-induced cleavage of the chromogenic substrate (S2238) by the peptide inhibitors.

Peptide ID	Peptide sequence (NH_2_-P3-P2-P1-P1′-CONH_2_)	K_i_ (µM)
1	D-Phe-Pro-D-Arg-Ala	16.64±0.8
2	D-Phe-Pro-D-Arg-D-Ala	2.06±0.04
3	D-Phe-Pro-D-Arg-Gly	5.9±0.2
4	D-Phe-Pro-D-Arg-Arg	91.1±8.2
5	D-Phe-Pro-D-Arg-Lys	66.56±6.5
6	D-Phe-Pro-D-Arg-Glu	555.7±89.6
7	D-Phe-Pro-D-Arg-His	122.1±9.5
8	D-Phe-Pro-D-Arg-Phe	250±20
9	D-Phe-Pro-D-Arg-Pro	89.5±5.5
10	D-Phe-Pro-D-Arg-D-Pro	489.26±112.9
11	D-Phe-Pro-D-Arg-Trp	65.55±7.2
12	D-Phe-Pro-D-Arg-Tyr	50.5±3.4
13	D-Phe-Pro-D-Arg-Val	56.32±1.5
14	D-Phe-Pro-D-Arg-D-Val	2.17±0.6
15	D-Phe-Pro-D-Arg-Thr	12.5±0.35
**16 (fPrt)**	**D-Phe-Pro-D-Arg-D-Thr**	**0.92±0.08**
17	D-Phe-Pro-D-Arg-Ser	17.06±0.8
18	D-Phe-Pro-D-Arg-D-Ser	12.3±0.08
**19 (fPrC)**	**D-Phe-Pro-D-Arg-Cys**	**16.50±1.50**
20	D-Phe-Pro-D-Arg-D-Cys	2.4±0.05
21	D-Phe-Pro-D-Arg-Gln	43.06±2.6
22	D-Phe-Pro-D-Arg-D-Gln	19.31±0.6
**23 (fPrI)**	**D-Phe-Pro-D-Arg-Ile**	**7.7±0.73**
24	D-Phe-Pro-D-Arg-D-Ile	7.44±0.5
25	D-Phe-Pro-D-Arg-Leu	20.6±4.5
26	D-Phe-Pro-D-Arg-D-Leu	4.13±0.24
27	D-Phe-Pro-D-Arg-Thi	8.16±0.35
28	D-Phe-Pro-D-Arg-Met	37.2±2.5

Virtual screening data supported a model of interaction between thrombin and peptide ligand in which the amino acid at position P1′ would make a relatively significant contribution to the free energy of interaction. Among the selected lead tetrapeptides (Table 2), the calculated free energy of interaction suggested tighter binding for those compounds with either the d-isomer of some of the polar uncharged amino acids (d-Gln, d-Cys, and d-Ser) or the somewhat unexpected l-Met or l-Thienylalanine (l-Thi) in P1′. An intermediate group of compounds comprised those containing the polar uncharged d-Thr, l-Ser or l-Gln as the terminal residue, while charged (l-Glu and l-Arg) and bulky (l-Ile, l-Phe, l-Trp, l-His) P1′ moieties ranked closely in a third group of putative binders. Considering the wide chemical space covered by the P1′ residues in these lead peptides, the only general SAR trend that could be observed is that, whenever both isomers of a given amino acid were present at this position, the d-isoform was predicted to have higher affinity for thrombin than the l counterpart (Table 2).

### Kinetics of thrombin inhibition by the synthetic peptides

The inhibitory efficiency of the designed peptides against bovine thrombin was evaluated by determining their inhibitory constant. The K_i_ values (Table 2) for the tetrapeptide inhibitors from the d-Phe-Pro-d-Arg-P1′-CONH_2_ series span almost 3 orders of magnitude. The experimental data confirmed the predicted SAR for the P1′ position from the docking experiments (Table 2; see above), suggesting that the interaction between the amino acid at the P1′ position and the S1′ pocket in thrombin is very selective. The preferred amino acids at the P1′ position belonged to the small hydrophobic (Ala, Gly, and d-Val) or polar uncharged groups (l- or d-Ser, Cys, Thr), with K_i_ values below 18 µM. Exceptions to this rule were observed for Ile, d-Leu and the unnatural amino acid l-Thi, with a K_i_ of approximately 8 µM.

The docking experiments predicted that the lead compounds with the d-isomer of Ser, Thr, Cys, Ala and Gln at P1′ were more efficient inhibitors that their l-amino acid-containing variants. This was verified experimentally, reaching its maximum expression in the case of Thr, where the d-isomer displayed a nearly 15-fold lower K_i_ than its l- counterpart (Table 2).

**Figure 2:**
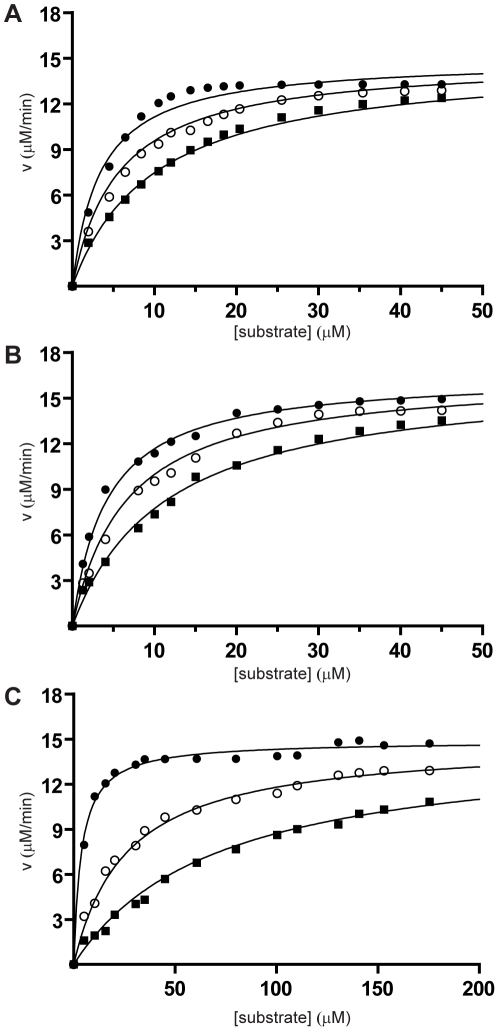
Inhibition of amidolytic activity of α-thrombin by peptide inhibitors. Cleavage of a chromogenic substrate (S2238) by bovine α-thrombin in the absence (•) and in the presence (○ - 5 µM; ▪ - 15 µM) of the tetrapeptides fPrI (A), fPrC (B) and fPrt (C). Data correspond to a representative set of peptide concentrations of at least three independent experiments. The derived K_M_ (3.65±0.3 µM) and V_max_ (15.17±0.18 µM/min) values for the reaction of bovine α-thrombin towards the S2238 substrate are in good agreement with the previously published kinetics parameters [35].

**Figure 3:**
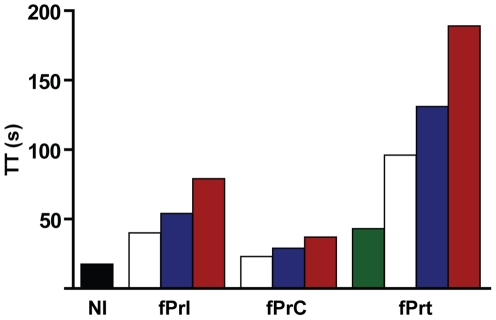
Prolongation of thrombin time by peptide inhibitors. Human plasma thrombin times were measured in the absence of inhibitor (NI) and in the presence of 0.10 mM (green bar) 0.25 mM (white bars), 0.5 mM (blue bars) or 1 mM (red bars) of the indicated tetrapeptide.

The three lead tetrapeptides (fPrI, fPrC and fPrt; Figures 1 and 2) that were also characterized structurally were found to be potent competitive thrombin inhibitors *in vitro* (Table 2). Furthermore, these peptides prolonged thrombin time (TT) in a dose-dependent manner (Figure 3), with relative activities that correlated well with their observed inhibition efficiency towards thrombin.

### Resistance to proteolytic cleavage

**Figure 4:**
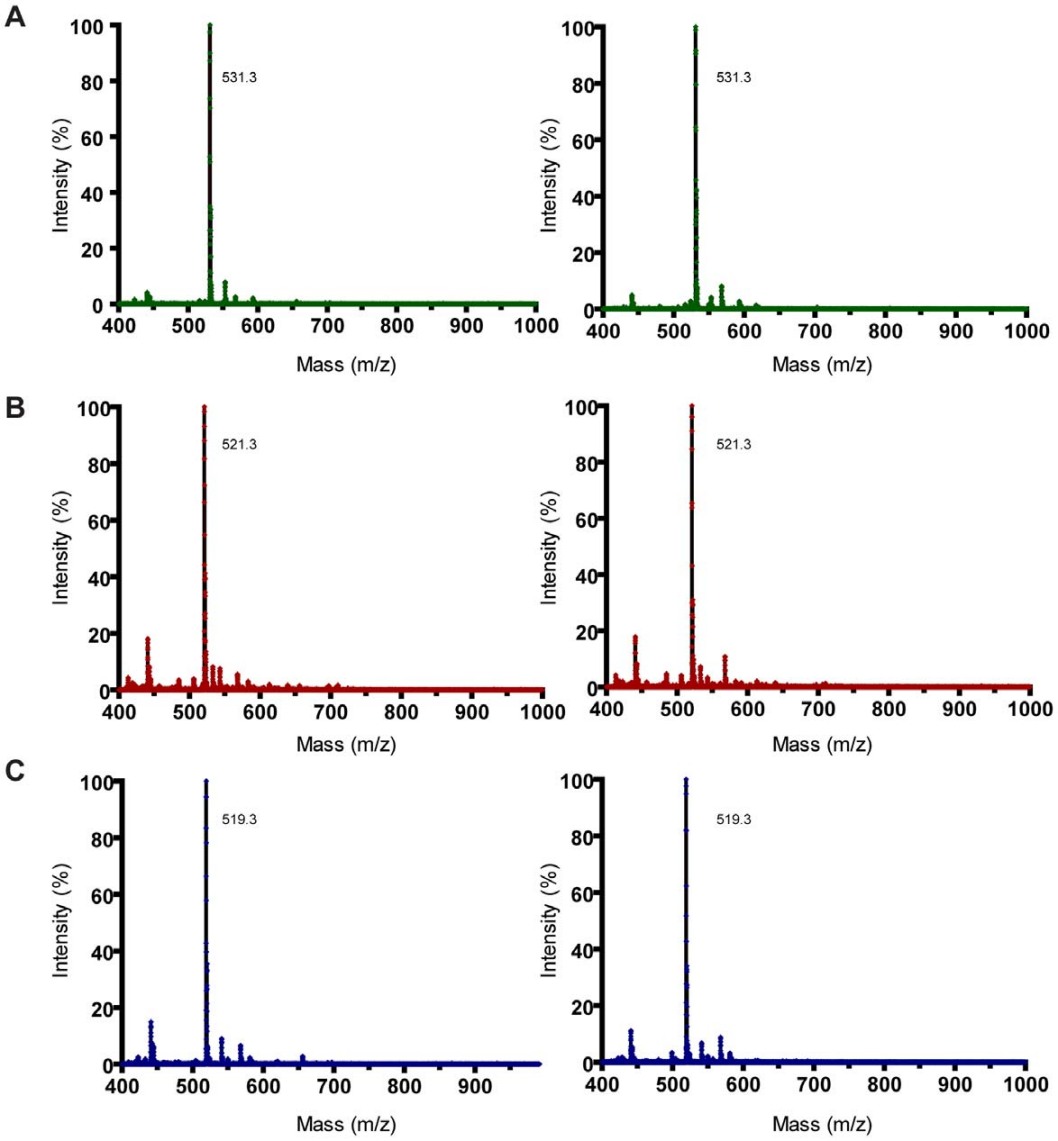
Stability of peptide inhibitors against thrombin hydrolysis. Mass spectrometry analysis of fPrI (A), fPrC (B), and fPrt (C) after incubation for 24 h at room temperature in the absence (left) or in the presence (right) of thrombin. The common putative cleavage product d-Phe-Pro-d-Arg with a molecular mass of 418.5 Da could not be identified in any of the proteinase-containing samples.

The three structurally characterized inhibitors were found to be stable to cleavage by thrombin, as no proteolytic fragments could be identified by mass spectrometry upon 24 h incubation with the enzyme at room temperature (Figure 4), in good agreement with their observed binding mode in the experimental crystallographic structures (see below).

### Selectivity for thrombin

The three structurally characterized peptide inhibitors display a higher selectivity for α-thrombin than for factor Xa or trypsin (Table 3). The best thrombin inhibitor, fPrt, is 420-fold and 110-fold more selective for thrombin than for trypsin or factor Xa, respectively. While fPrI is essentially unable to inhibit factor Xa *in vitro*, it displays a considerably more modest selectivity for thrombin versus trypsin (12-fold). Of the three tetrapeptides, fPrC was found to be the least selective, displaying only 3- or 20-fold selectivity towards both factor Xa or trypsin, respectively.

**Table 3:** Inhibition of factor Xa and trypsin by tetrapeptide inhibitors.

	K_i_ (µM)	
	factor Xa	trypsin
**fPrt**	103.06±1.44	388.74±5.5
**fPrC**	41.40±2.6	377.13±3.6
**fPrI**	7,300±11.5	90.2±2.8

### Structure of unliganded human α-thrombin

**Figure 5:**
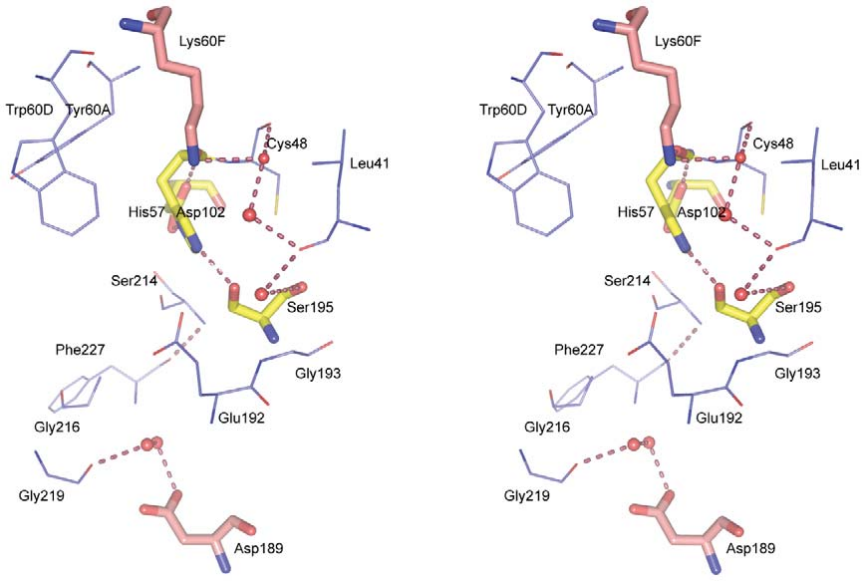
Stereo view of the active-site region of unliganded human α-thrombin. Thrombin residues are represented as thin lines (carbon depicted in cyan, oxygen in red, nitrogen in blue and sulfur in yellow). The side chains of Lys60F and Asp189 (carbon represented in pink, other elements as above), and of Asp102, His57 and Ser195 (carbon represented in yellow, other elements as above) are shown as stick models.

The structural model of unliganded human α-thrombin here reported (Figure 5) is strikingly similar to those of the proteinase in complex with small molecule inhibitors, with minor deviations in surface residues. Superposition of the heavy chain residues of unliganded α-thrombin with the equivalent residues of the thrombin∶PPACK complex [34] results in a r.m.s.d. of 0.39 Å for 248 aligned Cα atoms. Notably, the loops surrounding the active site preserve closely the conformation observed in the thrombin∶PPACK complex, except for loop 147 which is disordered in our model. There are also no evident distortions induced by crystal packing.

### Structure of thrombin-inhibitor complexes

**Figure 6:**
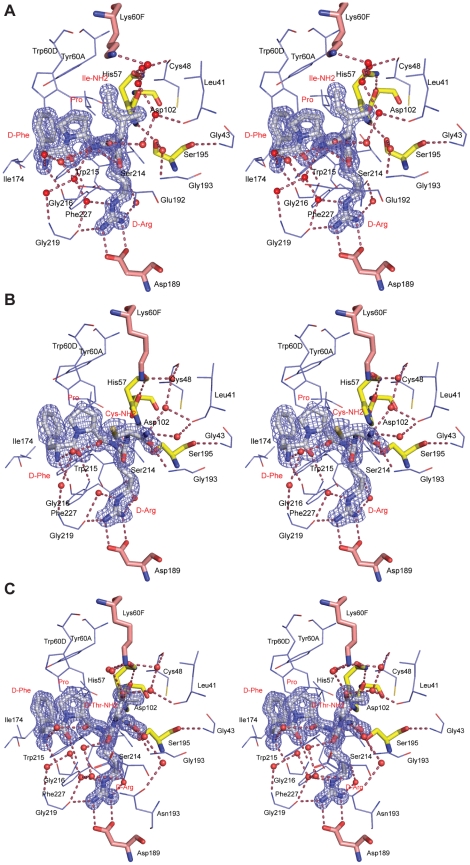
Stereo view of the active-site region of human α-thrombin in complex with the peptide inhibitors fPrI (A), fPrC (B) and fPrt (C). Thrombin residues establishing hydrogen bonds (red dashed lines) or hydrophobic contacts with the inhibitor are represented as thin lines (carbon depicted in cyan, oxygen in red, nitrogen in blue and sulfur in yellow). The side chains of Lys60F and Asp189 (carbon represented in pink, other elements as above), of Asp102, His57 and Ser195 (carbon represented in yellow, other elements as above) and the inhibitors (carbon is depicted in white, other elements as above) are shown as stick models. The electron density map (2Fobs-Fcalc) of the inhibitors is contoured at 1.5σ for fPrI and fPrt and at 1.0σ for fPrC. Inhibitor residues and selected thrombin side chains are labeled in red and in black, respectively.

The three-dimensional structures of three complexes of human α-thrombin with peptide inhibitors (general sequence d-Phe-Pro-d-Arg-P1′-CONH_2_ with l-isoleucine (fPrI), l-cysteine (fPrC) or d-threonine (fPrt) at the P1′ position) were determined by X-ray crystallography (Figure 6). The structure of the proteinase in all the complexes is very similar to that of the unliganded enzyme (248 Cα atoms of the heavy chain of thrombin can be aligned with a r.m.s.d. of 0.22, 0.17 and 0.12 Å for fPrI, fPrC and fPrt, respectively), with minor changes mostly in the side chain conformation of specific residues (Figure 5 and 6).

All the inhibitors bind in a substrate-like orientation to the active site of the enzyme, forming an antiparallel β-sheet with the Ser214-Gly216 segment of α-thrombin (Figure 6). Considering the invariable portion of the inhibitors, d-Phe and Pro are well known to be the preferred residues at positions P3 and P2, respectively [34]. The amine and carbonyl groups of d-Phe establish hydrogen bonds with Gly216 O and Gly216 N, respectively, while its aromatic side chain makes a stacking interaction with the indole group of Trp215, slotting between the side chains of Leu99 and Ile174. There is also a water-mediated contact between the N-terminal of the inhibitors and the main chain nitrogen of Gly219. The proline residue at position P2 establishes Van der Waals interactions with the side chains of Tyr60A and Trp60D, as well as with those of Leu99 and His57. In the thrombin∶fPrI complex the proline ring has a different puckering with concomitant adjustment of the positions of Trp60D and Tyr60A side chains (Figure 6 A).

The following P1 d-Arg residue displays polar interactions between its main chain nitrogen and the carbonyl oxygen of Ser214. The formation of this hydrogen bond, which is present in thrombin-PPACK, is suggested to play an important role in the generation of tetrahedral transition states in protease-substrate complexes [34]. Furthermore, the d-Arg side chain extends into the S1 pocket and the guanidinium group is hydrogen bonded to Asp189 (P1 NH2 - Asp189 OD1, P1 NH1 - Asp189 OD2) and to Gly219 (P1 NH1 - Gly219 O). There are also water-mediated hydrogen bonds connecting d-Arg NE and NH_2_ to the carbonyl oxygen of Gly219 and Phe227, respectively. In the fPrt complex, the carbonyl oxygen of d-Arg (P1) establishes water-mediated interactions with thrombin residues Glu192 (Glu192 N) and Gly219 (Gly219 O; Figure 6 C). These interactions are absent both in fPrI and fPrC complexes.

The contacts established by the d-Arg residue in position P1 with the enzyme backbone are similar to those observed for the l-isomer in other substrates and inhibitors (e.g. PPACK [34], MD-805/argotraban [9] and SDZ229-357 [35]. However, the presence of d-Arg at this position also results in the upstream residues sitting deeper in the S2 and S3 pockets, thereby increasing the distance between the Ser195 side chain hydroxyl and the P1 carbonyl carbon of the inhibitors (3.69 Å, 2.85 Å and 2.96 Å for fPrI, fPrC and fPrt, respectively; Table 4). Replacing the P1 residue with other variants of arginine such as β-homo-arginine (in hirulog3 [25]) or N-α-methyl-arginine (in I-11 [36]) was shown to improve resistance to thrombin hydrolysis. In all cases, the putative scissile bond becomes less accessible to the active site nucleophile. The TH146 (rOicPGF) [18] and FM19 (rOicPaF(*p*-Me)) [17] analogs of RPPGF, the angiotensin-converting enzyme breakdown product of bradykinin, inhibit thrombin in a retro-binding orientation inserting a d-Arg residue in the S1 specificity site in a similar way to that observed in our complexes, although without occupying the P1′ subsite.

**Table 4:** Distance between the catalytic Ser195 residue of thrombin and the P1 residue carbonyl carbon in thrombin-inhibitor complexes.

Thrombin complex	Ser195 OG - P1 C distance (Å)	Comments and References
**Thrombin : PPACK**	1.42	electrophilic carbonyl inhibitor (P1 = Arg) - irreversible [34]
**Thrombin : APPA**	1.44	electrophilic carbonyl inhibitor (P1 = aminophenylpyruvate) - reversible [38]
**Thrombin : CVS95**	1.74	bivalent inhibitor (P1 = Arg, P1′ = α-ketoamide) - transition state analogue - potent inhibitor (K_i_ = 1.01×10^−12^ M) [39]
**Thrombin : fPrI**	3.69	This work
**Thrombin : fPrC**	2.85	This work
**Thrombin : fPrt**	2.96	This work

Thrombin's Ser195 side chain occupies its canonical position (similar to that of the unliganded enzyme) in the fPrI complex, retaining the hydrogen bond between its OG and the side chain NE2 of His57 (Figure 6 A). However, in the fPrC and fPrt complexes, the Ser195 side chain is rotated away from the catalytic histidine residue, therefore disrupting the canonical hydrogen bond (Figure 6 B, C; Table 5), and interacts instead with the carbonyl oxygen of the P1′ residue. Hydrogen bonds between the inhibitor P1′ and thrombin Ser195 residues were observed in complexes of thrombin with non-covalent inhibitors Eoc-d-Phe-Pro-Abh and Cbz-Pro-Abh (P1′ N - Ser195 OG) [37] or CVS995 (P1′ O - Ser195 OG). We suggest that the catalytic triad disruption in thrombin∶fPrC and thrombin∶fPrt impairs the enzyme's hydrolytic ability, as proton abstraction by His57 NE2 and activation of Ser195 OG is no longer possible.

**Table 5:** Hydrogen bond distances between the catalytic His 57 NE2 and Ser195 OG in thrombin complexes.

Thrombin complex	Distance (Å)	Reference
**Unliganded thrombin**	2.80	This work
**Thrombin : APPA**	3.19	[38]
**Thrombin : PPACK**	2.90	[34]
**Thrombin : fPrt**	N.P.	This work
**Thrombin : fPrC**	N.P.	This work
**Thrombin : fPrI**	2.97	This work

N.P. - not present.

Substrate cleavage by thrombin is dependent on the formation of a tetrahedral transition state intermediate. Comparison of the interactions that thrombin establishes with fPrt, fPrC and fPrI with transition-state mimetic inhibitors can help explaining the resilience of the former compounds to proteolytic cleavage. Electrophilic carbonyl thrombin inhibitors such as PPACK [34] and APPA [38] form covalent transition-state-like analogs in complex with thrombin. The carbonyl carbon of their P1 residue (Arg in PPACK, aminophenylpyruvate in APPA) is in a tetrahedral configuration and covalently bound to Ser195 OG (Table 4). In these complexes the side chain of Ser195 is considerably rotated when compared to unliganded thrombin, however without disrupting the Ser195 OG - His57 NE2 hydrogen bond. The divalent potent thrombin inhibitor CVS995 is a competitive reversible inhibitor that displays an α-keto amide group at P1′ and forms a tetrahedral transition state with the active site serine and histidine residues [39]. Halomethylketones also form a covalent bond to His57 [6], [34].

The carbonyl oxygen atom of APPA and PPACK, located in the oxyanion hole, and the keto oxygen of CVS995 establish hydrogen bonds with the amide nitrogen atoms of Gly193 and Ser195 (Table 6). The interaction with Gly193 is present in the complexes thrombin∶fPrC and thrombin∶fPrt, whereas the latter interaction is absent. In the case of the thrombin∶fPrI complex Gly193N does not interact with the inhibitor, being instead indirectly bonded to Ser195 OG.

**Table 6:** Hydrogen bond distances between thrombin Gly193 N and Ser195 N and bound inhibitors.

Thrombin complex	Thrombin Atom	Inhibitor Atom	Distance (Å)	Reference
**Thrombin : APPA**	Gly193 N	O2	2.95	[38]
	Gly193 N	O1	3.23	
	Ser 195 N	O1	2.89	
**Thrombin : PPACK**	Gly193 N	P1 O	3.16	[34]
	Ser 195 N	P1 O	3.31	
**Thrombin : CVS995**	Gly193 N	P1 O	2.70	[39]
**Thrombin : fPrt**	Gly193 N	P1′ O	2.83	This work
**Thrombin : fPrC**	Gly193 N	P1′ O	3.24	This work
**Thrombin : fPrI**	Gly193 N	P1′ O	N.P.	This work

N.P. - not present.

The P1′ Cys of fPrC establishes Van der Waals contacts with Trp60D and is hydrogen bonded to Gly193 N and Ser195 OG through its carbonyl oxygen. Its terminal amide nitrogen (N2) also establishes solvent-mediated interactions with Leu41 O, Cys58 O and Lys60F NZ.

When d-Thr is found in the P1′ position (fPrt), the inhibitor is stabilized by water-mediated hydrogen bonds between its OG and Lys60F NZ, Leu41 O and Cys58 O and between its terminal amide nitrogen and Asn143 OD1 and Trp141 O. Additionally, fPrt interacts directly with thrombin through hydrogen bonds between its d-Thr carbonyl oxygen and both Gly193 N and the hydroxyl group of the catalytic Ser195. A d-Thr N - His57 NE2 interaction is observed as well, resembling that found in complexes of thrombin with active site inhibitors with benzothiazole [40] and ketothiazole [41] or with bivalent inhibitors with α-keto-amide [39] or 2-(4-aminobutyl)-hydrazyde (Abh) [37] at the P1′ position. Furthermore, d-Thr makes hydrophobic contacts with Trp60D, His57 and with the Cys42-Cys58 disulfide bond. All these interactions could be maintained in the case of d-Val at position P1′, except for the solvent-mediated contacts established by the side chain hydroxyl group of threonine, possibly accounting for the 2-fold lower K_i_ displayed by fPrt (Table 2). Finally, in the fPrI complex, the carbonyl oxygen of the isoleucine residue in position P1′ establishes water-mediated hydrogen bonds with Leu41 O and Cys48 O, while its terminal amide nitrogen interacts directly with the carbonyl oxygen of the P2 proline residue and the Glu192 OE2 via a solvent molecule. Furthermore, the P1′ isoleucine side chain also makes hydrophobic contacts with Trp60D and His57. It is conceivable that the similar-sized d-Leu side chain could establish equivalent contacts with the proteinase, as indicated by the comparable K_i_ determined for both peptides (Table 2).

The side chain of Lys60F was proposed to limit the size of thrombin's S1′ pocket contributing to the frequent occurrence of small residues (such as glycine and serine in thrombin-activated platelet receptors) at this position in natural protein substrates. In the thrombin∶fPrt and thrombin∶fPrC complexes, the side chain of Lys60F is found in an extended conformation similar to that observed in the thrombin-PPACK model [34] and in the unliganded enzyme. However, in the thrombin-fPrI complex, accommodation of the isoleucine in position P1′ implies the displacement of Lys60F, which is found in a different conformation similar to that observed in complexes of thrombin with inhibitors containing benzothiazole groups [40], [41], or bulky residues (Nle or Thi) at the P1′ position [42]. In addition, the terminal amide nitrogen of Ile points towards Trp60D, which moves further away from the catalytic site.

The atomic detail of the experimental three-dimensional structures provides a molecular explanation for the relative binding affinity and resistance to proteolysis of the designed compounds. The now identified peptidic direct thrombin inhibitors represent a novel pharmacophore scaffold for developing new antithrombotic agents by exploring the conformations imposed by the d-stereochemistry of the amino acids at positions P1 and P1′.
